# Adaptive heading correction for an industrial heavy-duty omnidirectional robot

**DOI:** 10.1038/s41598-022-24270-x

**Published:** 2022-11-15

**Authors:** Rocco Galati, Giacomo Mantriota, Giulio Reina

**Affiliations:** Department of Mechanics, Mathematics, and Management, Polytechnic of Bari, via Orabona 4, 70126 Bari, Italy

**Keywords:** Engineering, Mechanical engineering

## Abstract

The paper deals with the design and testing of a robot for industrial applications featuring omnidirectionality thanks to the use of mecanum wheels. While this architecture provides remarkable manoeuvrability in narrow or cluttered spaces, it has some drawbacks that limit its widespread deployment in practice, especially for heavy-duty and long-duration tasks. As an example, the variability in the mecanum wheel rolling radius leads to undesired dynamic ill-effects, such as slippage and vibrations that affect the accuracy of pose estimation and tracking control systems. Drawing on the modeling of the kinematic and dynamic behaviour of the robot, these effects have been tackled within an adaptive estimation framework that adjusts the robot control system based on the properties of the surface being traversed. The proposed approach has been validated in experimental tests using a physical prototype operating in real industrial settings.

## Introduction

In recent years, mobile robots have been increasingly used in various applications of logistics and supply chain management^1–3^. Several examples have been demonstrated to optimize and improve industrial processes, drawing attention from both academy and industry^4–6^. Many companies are trying to speed up their production processes by applying just-in-time (JIT) strategies with the aim of increasing efficiency and reducing waste by receiving goods only as needed in the production process by reducing inventory costs^7^. As a result of the adoption of new technologies in many industrial plants, the efficiency of the production processes has increased causing an overloading of the logistics area as a consequence. For this reason, a series of unmanned ground vehicles (UGV) have been designed to operate as logistic robots for picking^8^, palletizing^9^, and handling^10^ products. Omnidirectional robotic platforms have attracted much attention since they are capable of driving in any direction by minimizing the total path between two points and they are suitable for compact and narrow industrial spaces^11^.

Omnidirectional vehicles have been proposed^12–14^ with varying wheels and chassis configurations, the most important of which are based on three-wheel and four-wheel schemes^15^. Indeed, the number of mecanum wheels affects the power consumption^16,17^: a four wheeled system allows to harness the motors power up to 50%, when translating along the axis of a wheel, and to 71%, when translating 45 deg from an axis of a wheel, while a three wheeled configuration enables the system to use up to 47% of the total motors power when translating along the axis of a wheel and up to 68%, when translating 30 deg from an axis of a wheel^18^.

**Figure 1:**
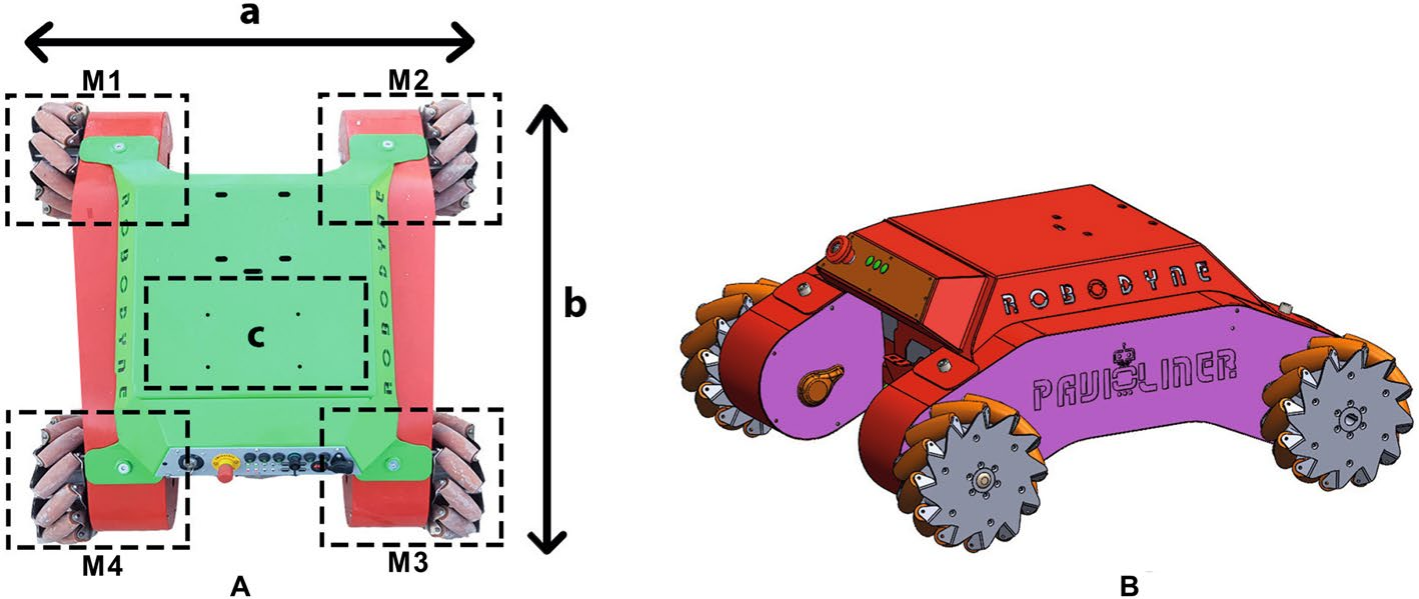
The omnidirectional vehicle used for this research study: (**A**) top view of the real robot, (**B**) isometric view of the 3D rendering.

Most of the omnidirectional robots proposed in the literature have been used for research purposes or lightweight applications^19^ where no payload^20^ or limited payload^21^ is present, whereas no heavy duty industrial example has been discussed.

One common drawback of all vehicles using mecanum wheels is that they are affected by random slippage^22^ and high-speed vibration^23^ that lead to position errors and energy dissipation problems^24,25^. This is especially true for robots that are required to carry large loads. In this research, an omnidirectional robot is presented designed to be employed for heavy duty applications in industrial sites or service tasks that feature significant payload and long-duration operations. Critical issues are addressed for the practical deployment of the system, including variability in the wheel radius and accurate pose estimation even in the presence of unpredictable slip occurrences incurred by the mecanum wheels. A robust and adaptive trajectory tracking system is presented as well that adjusts automatically the parameters of the control system according to the specific supporting surface.

## Materials and methods

### Vehicle overview

The omnidirectional vehicle used for this research, named Omnibot, is showed in Fig. 1. Ominibot is based on a rectangular plan having an overall length and width of, respectively, $b=1012$ mm and $a=1038$ mm that provide a large supporting base. At each of the four corners, an assembly made up of a mecanum wheel, a gearbox with reduction ratio $i=30$, a 500 W brushed DC motor with a speed of 3000 rpm and a torque of 35 Nm, and a 1024 pulse optical encoder is placed to provide an efficient and robust locomotion system. Motors are controlled by a total of four drivers, featuring a high-performance 32-bit microcomputer and quadrature encoder input to perform advanced motion control algorithms in both open-loop and closed-loop modes for speed and position. In order to enable the vehicle to increase its working autonomy, a 24 VDC 200 Ah AGM battery pack has been installed in *c* to provide a maximum output power of 4.8 kW with a 350 A battery isolator switch based on a high-load solenoid. Each mecanum wheel has a vertical load capacity of 250 kg and the metal frame of the robot has a total payload of 1000 kg to allow the use of large and heavy equipment in industrial environments. The robot has been completely custom built starting from CAD design, and all the parts have been manufactured by using laser cut and bending machines. Table 1 summarizes the main mechanical specifications.

**Table 1:** Omnibot main technical specifications.

Omnibot specifications	Value
Number of motors	4
Overall dimensions	1012 $\times$ 1038 mm
Max velocity (any direction)	1 m/s
Max Payload	1000 kg
Output power	4800 W
Vehicle weight	200 kg

### Omnidrivability

**Figure 2:**
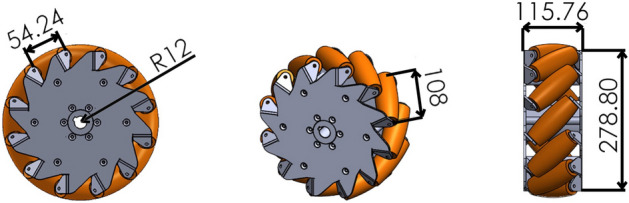
Details of the mecanum wheels: (**A**) side view; (**B**) isometric view, and (**C**) front view where it is possible to see the roller details. All measurements are in mm.

Omnibot fulfills holonomy by adopting four mecanum wheels, whose main specifications have been collected in Table 2. Both outer plates with a maximum diameter of about 278 mm are made of stainless steel and feature twelve teeth with a pitch of about 54 mm placed on their outer circumference and bent at 45 deg. Each pair of outer and inner teeth is used to mount a passive soft nylon roller with a length of 108 mm for a total of twelve rollers. Figure 2 reports three different views and the overall dimensions of a single wheel. The angled rollers translate a portion of the force in the rotational direction of the wheel to a force that is normal to the wheel direction. Depending on each individual wheel direction and speed, the resulting combination of all these forces allow the vehicle to move in any direction.

**Table 2:** Mecanum wheel specifications.

Mecanum wheel specifications	Value
Number of rollers	12
Roller angle	$45^\circ$
Diameter of mecanum wheel	280 mm
Diameter of roller at its center	46 mm
Diameter of roller at the extremities	34 mm
Wheel mass	15 kg

### Control and acquisition system

**Figure 3:**
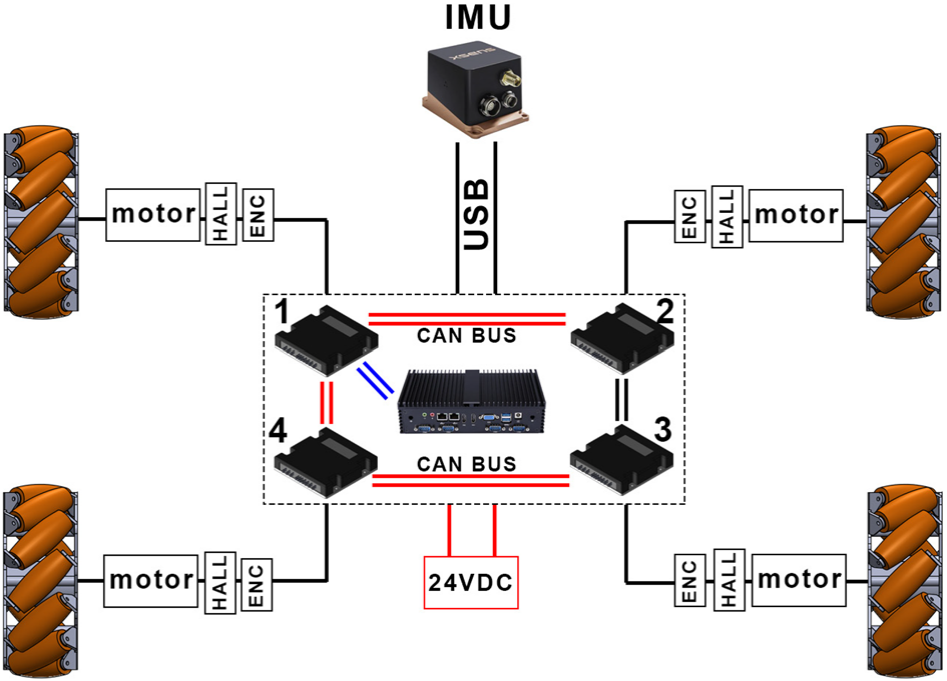
The control architecture of Omnibot.

The control architecture of Ominibot is reported in Fig. 3. Four power drivers are used over a CAN Bus network where the controller for the front left wheel is the master and all the other ones are set as slaves. The main operating system runs on an industrial computer with an Intel i7 CPU and powered by ROS featuring also three USB 3.1 ports useful to connect different kind of sensors such as the Inertial Measurement Unit, XSens Mti-300, mounted on the front bumper of the vehicle in order to detect velocities and accelerations. Note that each motor is equipped with a Hall sensor to read the electrical current.

## Kinematics and dynamics modeling

### Inverse kinematics

**Figure 4:**
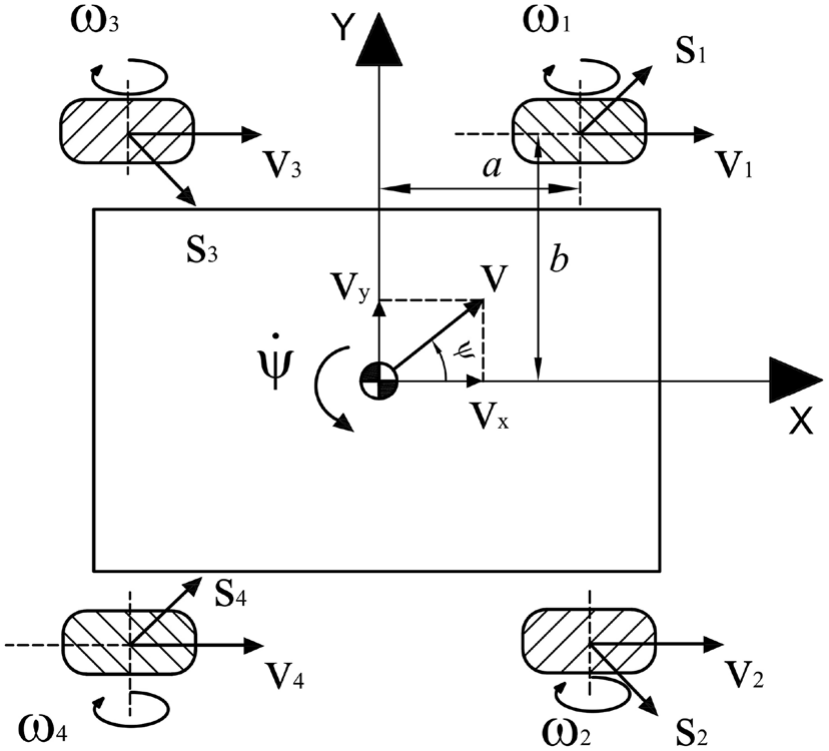
Movement vector and coordinate system of mobile four-wheel drive vehicles.

With reference to Fig. 4, the inverse kinematic model of the omnidirectional vehicle can be derived from the knowledge of the velocity vector *V*, whose components along the $X-$ and $Y-$ axes are, respectively,:

1 $$\begin{aligned} v_x= v cos\psi \end{aligned}$$

2 $$\begin{aligned} v_y= v sin\psi \end{aligned}$$

where $\psi$ identifies the orientation of the vehicle while the angular velocity is defined by ${\dot{\psi }}$. Important geometric parameters are the mecanum wheel radius *r*, the distance *a* between the body and the wheel center along the *x* axis and by the length of *b* which is the distance between the body and the wheel center along the *y* axis:

3 $$\begin{aligned} a_i= \{a, a, -a, -a\} \end{aligned}$$

4 $$\begin{aligned} b_i= \{b, -b, b, -b\} \end{aligned}$$

where *i*: 1,2,3,4 represents each mecanum wheel. The linear velocity vector and the velocity of mecanum nylon roller direction for each wheel are indicated by $v_i$ and $s_i$, respectively. Tilted angle $\gamma$ between *v* and *s* is $45^\circ$ and represents the mecanum roller angle:

5 $$\begin{aligned} \gamma _i = \left\{ \frac{\pi }{4}, -\frac{\pi }{4}, -\frac{\pi }{4}, \frac{\pi }{4}\right\} \end{aligned}$$

The velocity vector equation of the vehicle towards the two coordinate system components can be calculated by:

6 $$\begin{aligned} v_i + s_icos\gamma _i= v_x - b_i {\dot{\psi }} \end{aligned}$$

7 $$\begin{aligned} s_i sin\gamma _i= v_y + a_i {\dot{\psi }} \end{aligned}$$

By using equations (6) and (7), it is possible to derive all the linear velocities associated to each mecanum wheel:

8 $$\begin{aligned} v_1= v_x - \frac{v_y}{\tan \gamma _1} - \frac{a{{\dot{\psi }}}}{\tan \gamma _1} - b{{\dot{\psi }}} \end{aligned}$$

9 $$\begin{aligned} v_2= v_x - \frac{v_y}{\tan \gamma _2} - \frac{a{{\dot{\psi }}}}{\tan \gamma _2} - b{{\dot{\psi }}} \end{aligned}$$

10 $$\begin{aligned} v_3= v_x - \frac{v_y}{\tan \gamma _3} - \frac{a{{\dot{\psi }}}}{\tan \gamma _3} - b{{\dot{\psi }}} \end{aligned}$$

11 $$\begin{aligned} v_4= v_x - \frac{v_y}{\tan \gamma _4} - \frac{a{{\dot{\psi }}}}{\tan \gamma _4} - b{{\dot{\psi }}} \end{aligned}$$

Knowing that the wheel velocities can also be expressed as $v_i = {\dot{\psi }} r$, equations from (8) to (11) can be written in compact matrix form:

12 $$\begin{aligned} \begin{bmatrix} w_1 \\ w_2 \\ w_3 \\ w_4 \end{bmatrix} = \left( \frac{1}{r}\right) R \begin{bmatrix} v_x \\ v_y \\ {\dot{\psi }} \end{bmatrix} \end{aligned}$$

Equation (12) shows the mathematical model of the inverse kinematic to obtain the angular velocities for the mecanum wheels by using as input the three components of the velocity $v_x$, $v_y$ and ${\dot{\psi }}$ where the matrix *R* is defined as follows:

13 $$\begin{aligned} R = \begin{bmatrix} 1, -\frac{1}{\tan \gamma _1}, -\frac{a}{\tan \gamma _1 + b} \\ 1, -\frac{1}{\tan \gamma _2}, -\frac{a}{\tan \gamma _2 + b} \\ 1, -\frac{1}{\tan \gamma _3}, -\frac{a}{\tan \gamma _3 + b} \\ 1, -\frac{1}{\tan \gamma _4}, -\frac{a}{\tan \gamma _4 + b} \end{bmatrix} \end{aligned}$$

By inverting the matrix *R* using the Moore-Penrose theorem, it is possible to obtain the direct kinematic model to retrieve $v_x$, $v_y$ and ${\dot{\psi }}$ given the wheel angular velocities:

14 $$\begin{aligned} \begin{bmatrix} v_x \\ v_y \\ {\dot{\psi }} \end{bmatrix} = \left( \frac{r}{4}\right) R^+ \begin{bmatrix} w_1 \\ w_2 \\ w_3 \\ w_4 \end{bmatrix} \end{aligned}$$

where $R^+$ is the pseudoinverse matrix of R.

### Rolling radius

An important aspect of the Omnibot kinematics refers to the knowledge of the wheel radius. By analyzing the mecanum wheel rotational motion, it is possible to observe that there is a constructive limit due to the presence of the nylon rollers that prevents the wheels from having a continuous contact surface and therefore to keep the radius of the wheel constant. This contributes to generate different tangential speeds for each individual wheel^26^. It is critical to address this problem in order to limit the wear of the rollers and to avoid unpredictable deviations from the intended path that are difficult to predict by standard odometry approaches. When one looks at the longitudinal middle section of the wheel, it is possible to obtain the radius change as a function of the wheel rotational angle. Since each mecanum wheel is made up of twelve rollers, the radius changes periodically every 30 deg ranging from its starting value up to the maximum measured value as it is showed in Table 3 where radius values for some intermediate wheel rotations are reported.

**Table 3:** Radius values for 5-deg steps of wheel rotation.

Position (deg)	Radius (m)
0	0.1524
5	0.1519
10	0.1504
15	0.1480
20	0.1504
25	0.1519
30	0.1524

**Figure 5:**
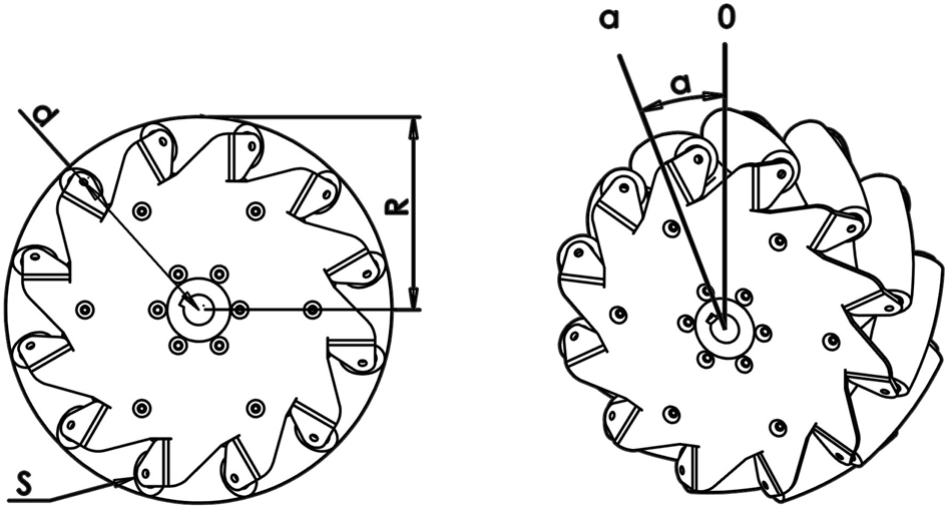
Radius variation for each mecanum wheel.

**Figure 6:**
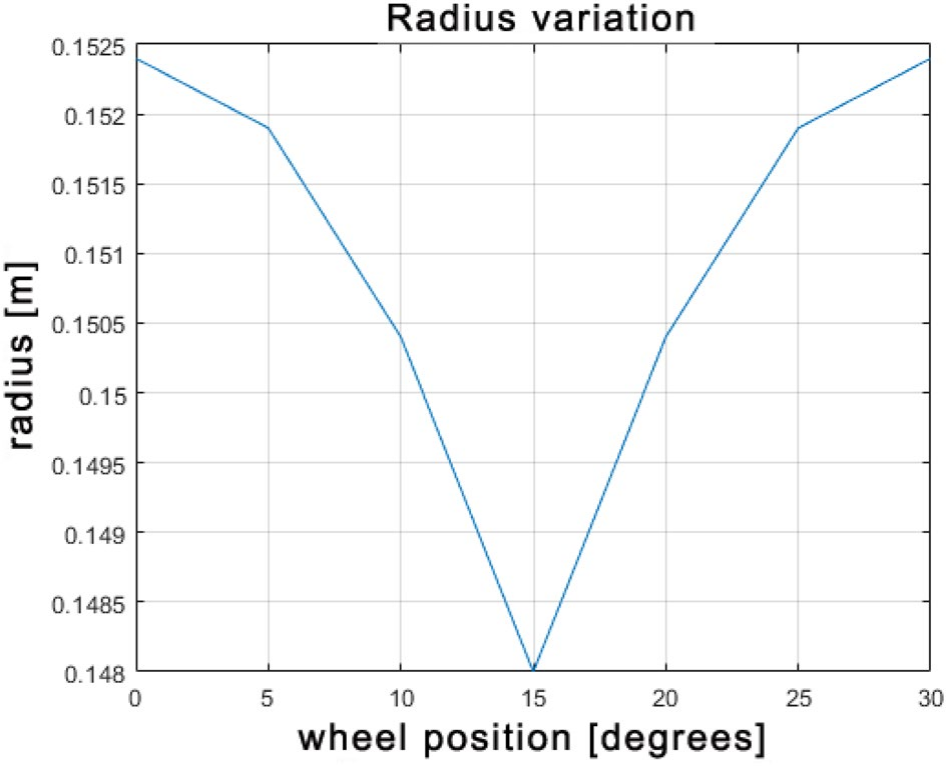
Radius variation against the wheel angular position.

As showed in Fig. 5, by using the relation to calculate the radius variation for each mecanum wheel position:

15 $$\begin{aligned} r(\alpha ) = s + d cos(\alpha ) \end{aligned}$$

where *s* is the roller radius, *d* is the distance between the roller and the wheel hub, $\alpha$ is the current angular position for each wheel, it is possible to improve the performance of the motion controller. Figure 6 shows the radius as a function of the wheel rotation. The radius variation affects not only the wear of the rollers but also the total path of the vehicle that becomes not predictable. This path error has been reduced by integrating the function of the radius by time in order to increase the accuracy of the total traveled distance.

### Wheel slipping

When a torque is applied to a mecanum wheel, it starts to rotate without slipping as long as the driving force, *F*, does not exceed the maximum static friction, $\mu$, i.e., $F < \mu F_z \sin (45)$. When the threshold is overcome, the wheel slips as it rolls, resulting in unpredictable deviations. Under the assumption of uniform weight distribution and a vehicle mass of 200 kg, a single wheel is subject to a quarter of the total vehicle weight *Gtot* (1962 N), i.e., $F_z=\frac{Gtot}{4}=490.5$ N. Friction values can be found in the literature, for example^27^, for most common surfaces. For example, $\mu _c = 0.5$ for industrial concrete floor and $\mu _a = 1$ for asphalt. Then, in order for a given wheel to perform a pure rolling motion, the following condition must be satisfied:

16 $$\begin{aligned} T \le T_{max}=\mu F_z \sin (45)r \end{aligned}$$

where *T* indicates the torque applied to the wheel, and $r = 0.15093$ m the average radius. The maximum torque that can be delivered by each drive motor is $T_{max} = 47$ Nm as obtained from the following formula:

17 $$\begin{aligned} T = \frac{60P}{2\pi RPM} \end{aligned}$$

being $P=500W$ and $RPM=3000/\tau =100$ rpm, where $\tau =30$ is the gearbox reduction ratio. By considering equation (17), it is also possible to introduce the relation between the mechanical torque, and the electric current drawn by each motor:

18 $$\begin{aligned} T = i\tau _t I \end{aligned}$$

where *i* is the gearbox reduction ratio, $\tau _t = 0.079$ Nm/A is the motor torque constant and *I* is the electric current drawn by each DC motor.

### Vertical dynamics

It can be observed that the variation of the wheel radius, as described in the previous section, forces the wheel to oscillate and the overall vehicle vibrational response can be considered as modulated by the interaction of the wheel with the specific ground surface^28^. Even if the angular position of all four mecanum wheels is identical when the vehicle starts to move, a perfect synchronization is very difficult during operations due to the slipping effects between each roller and the ground surface. Although the radius variation is independent of the ground surface, it is important to highlight that the vehicle vibration response is strictly related to the interactions between the mecanum wheels affected by their radius variation and the ground surface; it is therefore possible to find out a specific vehicle’s behavior by studying the power spectral density function. In linear systems, it exists a direct linear relationship between input and output. A vehicle system defined by its transfer function takes into account the input representing the terrain irregularities and generates an output representing the vibration of the vehicle^29^. In this case, the frequency response function can be defined as the ratio of the output to input under steady-state conditions.

In order to simplify the study of the vertical motion of Omnibot, a one-degree-of-freedom quarter vehicle model has been considered^30^. This assumption has been widely adopted in the literature for the analysis of many robots, e.g.^31,32^. Further studies^33^ have demonstrated how the theory of vibration of single-degree-of-freedom systems serves as one of the fundamental building blocks in the theory of vibration of discrete and continuous systems and the techniques developed for the analysis of single degree of freedom systems can be generalized to study discrete systems with multi-degree of freedom as well as continuous systems. Then, if it is possible to consider a simplified single-degree-of-freedom model for the vehicle, and the surface irregularity as input is defined in terms of displacement (or elevation of the surface profile) and the vibration of the sprung mass as output is measured in acceleration, then the modulus of the transfer function *H*(*f*)^27^, can be expressed as:

19 $$\begin{aligned} |(H(f)|=(2\pi f)^2\sqrt{\frac{1+\left( 2\zeta \frac{f}{f_n}\right) ^2}{\left[ 1-\left( \frac{f}{f_n}\right) ^2\right] ^2+\left[ 2\zeta \frac{f}{f_n}\right] ^2}} \end{aligned}$$

where and $\zeta =0.1$ is the damping ratio, *f* is the frequency of excitation and $f_n$ is the natural frequency of the system, which is approximately $f_n=38Hz$ for the considered vehicle as obtained experimentally by using an inertial sensor and an impact piezoelectric hammer with a load cell tip. When the transfer function of a specific system is known, then, it is possible to express the relation between the power spectral density of the input $S_g(f)$ and the power spectral density of the output $S_v(f)$ of the whole system as follows:

20 $$\begin{aligned} S_v(f)=|H(f)^2|S_g(f) \end{aligned}$$

This relation shows how the output power spectral density is associated to the input power spectral density through the square of the modulus of the transfer function. The power spectral density defines how the power of a signal is distributed over frequency and it is strictly correlated to the interaction between the ground surface and the mecanum wheels nylon rollers. The study of this important aspect allows finding the proper fingerprint for each ground surface.

## Omnibot pose estimation and trajectory tracking

**Figure 7:**
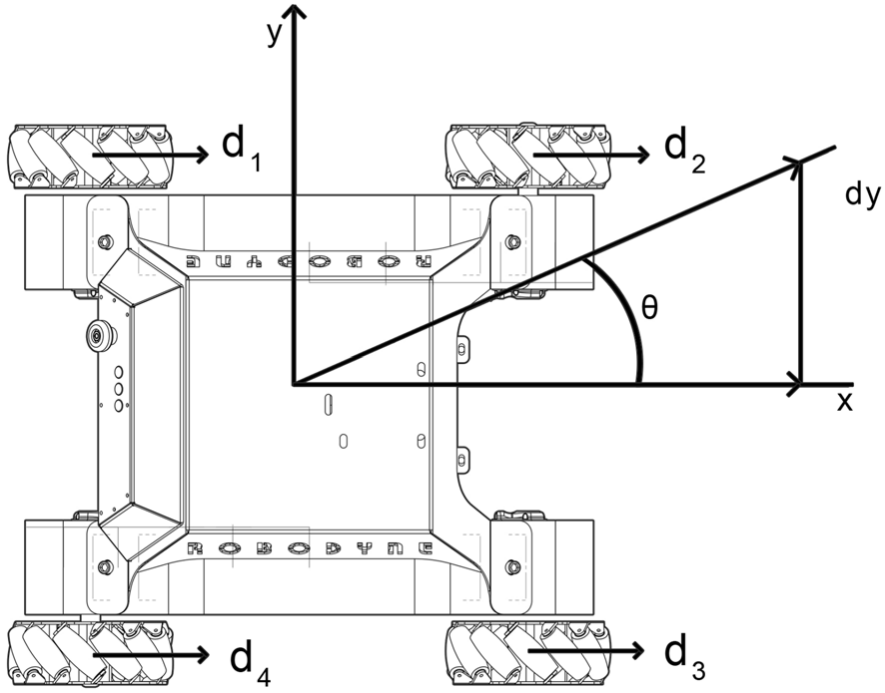
The straying angle caused by slipping episodes incurred by the mecanum wheels.

Since vehicles that employ mecanum wheels are able to rotate and move in narrow spaces, they are very suitable for industrial applications. However, the slipping effects on each roller can introduce a significant trajectory error both during straight motion and also during lateral displacements, and so it is difficult to retrieve the correct vehicle position. In order to better define the straying angle for the vehicle, it is possible to introduce a deviation angle $\theta$ which is the difference between the angle the vehicle should have and the current angle. As showed in Fig. 7, when the vehicle is commanded to move forward on a straight line, all the motors receive the same PWM commands and they start spinning with the same velocity and towards the same direction. The surface irregularities and the slipping effects lead to a lateral drift $d_y$, which can be defined as:

21 $$\begin{aligned} d_y=\left( \frac{1}{4}(d_1+d_2+d_3+d_4)\right) \tan \theta \end{aligned}$$

where $\theta$ is obtained by integrating the yaw rate measured by the on board inertial sensor. The resulting value of $d_y$ is strictly related to vehicle geometry and to the ground properties.

In order to improve the accuracy of the pose estimation system, a Kalman filter is adopted by using the readings coming from the gyroscope and the accelerometers included in the XSens Mti-300 inertial sensor despite the presence of significant errors in real time measurements^34^. Noisy accelerometers and gyro can be combined to obtain an accurate representation of unit’s orientation and position^35^. The used Kalman filter basically consists of two stages: during the first stage, a mathematical state model is applied to make a prediction about the system state while, during the second stage, this state prediction is compared to measured state values. The difference between the predicted and measured state is based on estimated noise and error in the system and measurements while a state estimation is generated as output. Finally, this output estimation is used in conjunction with the mathematical state model to predict the future state during the next time update, and the cycle begins again. By considering the inertial measurement unit’s output, the system state *x*(*t*) can be expressed as:

22 $$\begin{aligned} x(t) = \begin{bmatrix} p_x(t) \\ p_y(t) \\ v_x(t) \\ v_y(t) \\ a_x(t) \\ a_y(t) \end{bmatrix} \end{aligned}$$

where *x*(*t*) expresses the system state at time *t*, while $p_x(t)$ and $p_y(t)$ represent the position along the X and Y axes, respectively, $v_x(t)$ and $v_y(t)$ are the velocities in the x–y plane and $a_x(t)$ and $a_y(t)$ are the accelerations along the X and Y axis at time *t*. The Kalman filtering estimation operates through the prediction-correction cycle expressed as follows: Prediction:

23 $$\begin{aligned} \hat{x^-}_{t+1}= A_d\hat{x_t} + B_d u_t \end{aligned}$$

24 $$\begin{aligned} P_{t+1}^-= A_d P_k A_d^T + Q \end{aligned}$$

**Figure 8:**
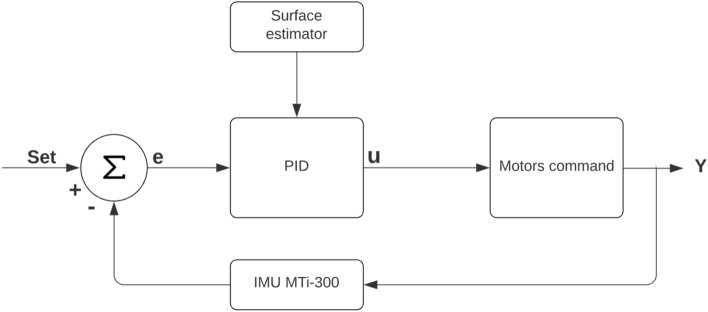
The PID controller used for the closed-loop system used to generate motors commands.

Correction:

25 $$\begin{aligned} K_{t+1}= P_{t+1}^- H_d^T (H_d P_{t+1}^- H_d^T + R)^-1 \end{aligned}$$

26 $$\begin{aligned} {\hat{x}}_{t+1}= \hat{x_{t+1}^-} + K_{t+1} (z_{t+1} - H_d \hat{x^-}_{t+1}) \end{aligned}$$

27 $$\begin{aligned} P_{t+1}= (I - K_{t+1} H_d) P_{t+1}^- \end{aligned}$$

where $\hat{x^-}_{t+1}$ is the predicted state vector, *A* and *B* are the state transition matrix, $P_{t+1}^-$ is the variance matrix for $\hat{x^-}_{t+1}$, $K_{t+1}$ is the gain matrix, ${\hat{x}}_{t+1}$ is the updated state vector, and $P_{t+1}$ is the updated error covariance estimation; the noise covariance of the process is expressed by *Q*, while *R* accounts for the uncertainty in the measurement and is set as $R = \sigma _{gyro}$. The updated position information from the Kalman filter, and, especially, the heading of the vehicle is used to feed a combination of proportional, integrative and derivative controls (PID) with the aim to keep the vehicle on its track while commanded to move straight forward, backward or laterally by generating the proper corrections for all four motors as reported in Fig. 8. Even if other advanced control strategies, such as model predictive control (MPC) and slide model control (SMC), have been used in the literature and are gaining significant attention from both industry and academia, for this specific application, PID control has been preferred since it requires less computational time, implementation efforts, and memory consumption given the hardware resources installed on board the vehicle to keep the system cost-effective and easy to produce on a large scale, as happens in conventional industrial scenarios. Typical final applications for Omnibot, like the automatic floor marking described later in the paper can be considered as non-delay dominant processes and so they are particularly suitable for a PID controller. The controller continuously calculates an error *e* as the difference between a desired set-point and the measured heading value calculated by the MTi-300 by applying a correction. Equation (28) describes the behavior of the controller implemented for trajectory correction:

28 $$\begin{aligned} u = A_c K_p e + K_r\int e \,dt \ + B_c K_d \frac{de}{dt} \end{aligned}$$

where *u* is the measurement sent to the motors command algorithm, $K_p$ is the proportional gain while $K_r$ and $K_d$ are the integrative and derivative gains, respectively. The parameters $A_c$ and $B_c$ are generated by a surface classifier algorithm based on the analysis of the power spectral density (PSD) calculated over the vertical accelerations and motor currents: in particular, by measuring the different accelerations along the Z axis and the currents drawn by each motor, it is possible to detect the features of the ground surface where the vehicle is moving on. Experimental evidence shows how the magnitude of the trajectory error is strictly related to the interactions between the mecanum wheels rollers and the ground surface by resulting in a different straying angle. Depending on the surface characteristics, the surface estimator sets the value of $A_c$ and $B_c$ to improve the corrections over the proportional and derivative gains. The output *Y* includes all the parameters ($v_x$, $v_y$ and ${\dot{\psi }}$) needed to control the motors as specified in Eq. (14).

## Experimental results

**Figure 9:**
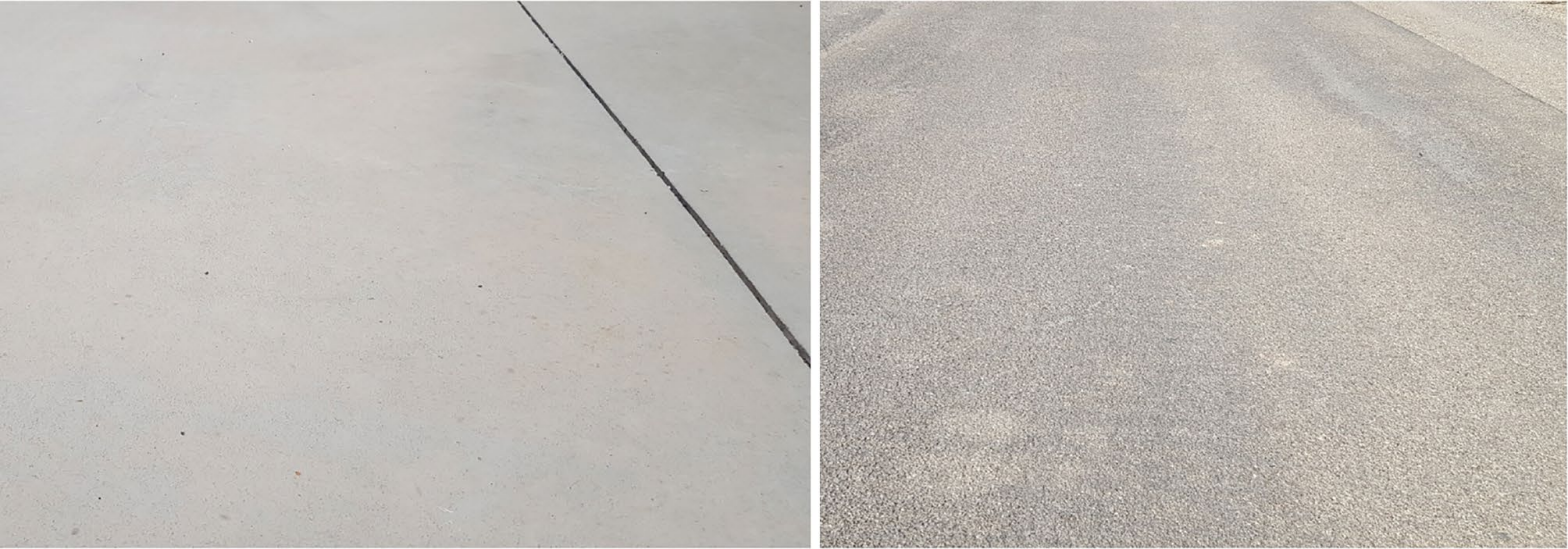
The surfaces used for this research study: concrete, on the left, and asphalt, on the right.

Different tests in a real industrial setting have been run in order to assess the performance and validate the adaptive control system. In the first test case, the vehicle has been commanded to follow two motion primitives, moving straight forward and sideways, over two different ground surfaces: industrial concrete floor and asphalt. The first one is made by a flat and leveled slab formed by concrete and it is designed to withstand heavy-duty vehicular traffic while its elastic modulus is 40 GPa and its stiffness module is 70 MPa. The asphalt surface is made by a bituminous granular mixtures used for public roads with a stiffness module of 140 MPa and an elastic modulus of 480 MPa. Sample patches for both the ground surfaces are showed in Fig. 9 and as it can be noted, the concrete surface is regular and free of debris while the asphalt surface is irregular and dirtier. No other surfaces have been considered since the proposed vehicle is intended to operate in warehouses, where concrete and asphalt are the two typical pavements.

**Figure 10:**
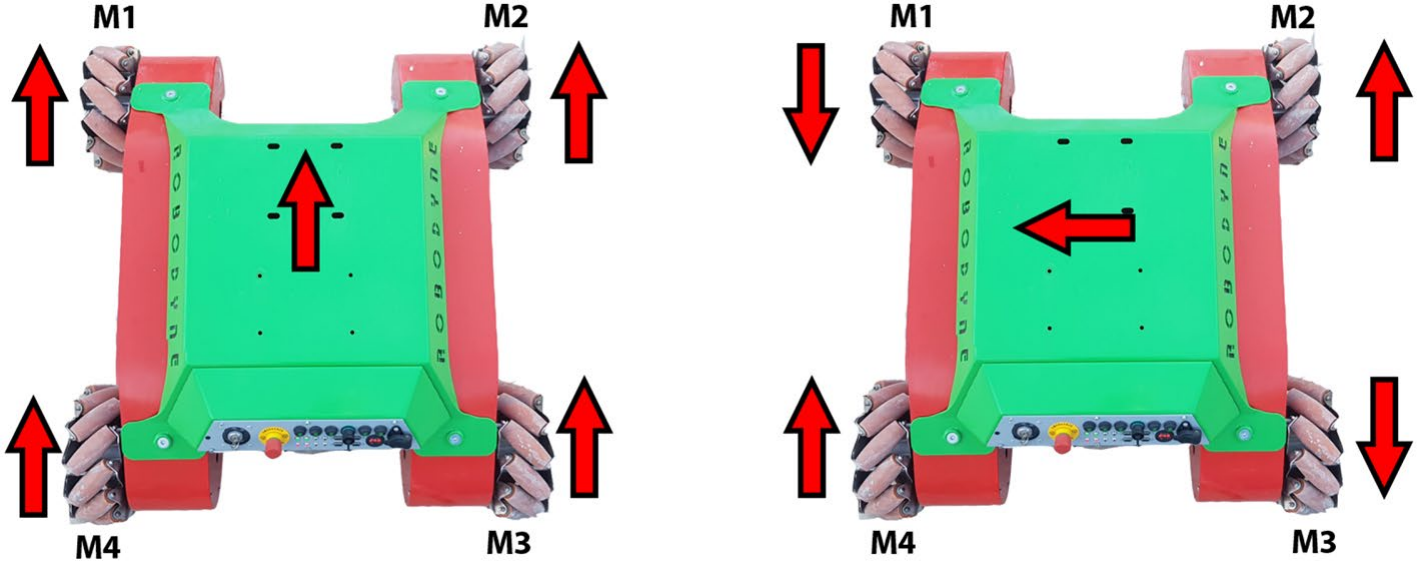
Wheel spinning configuration for the two motion primitives used for the tests.

**Figure 11:**
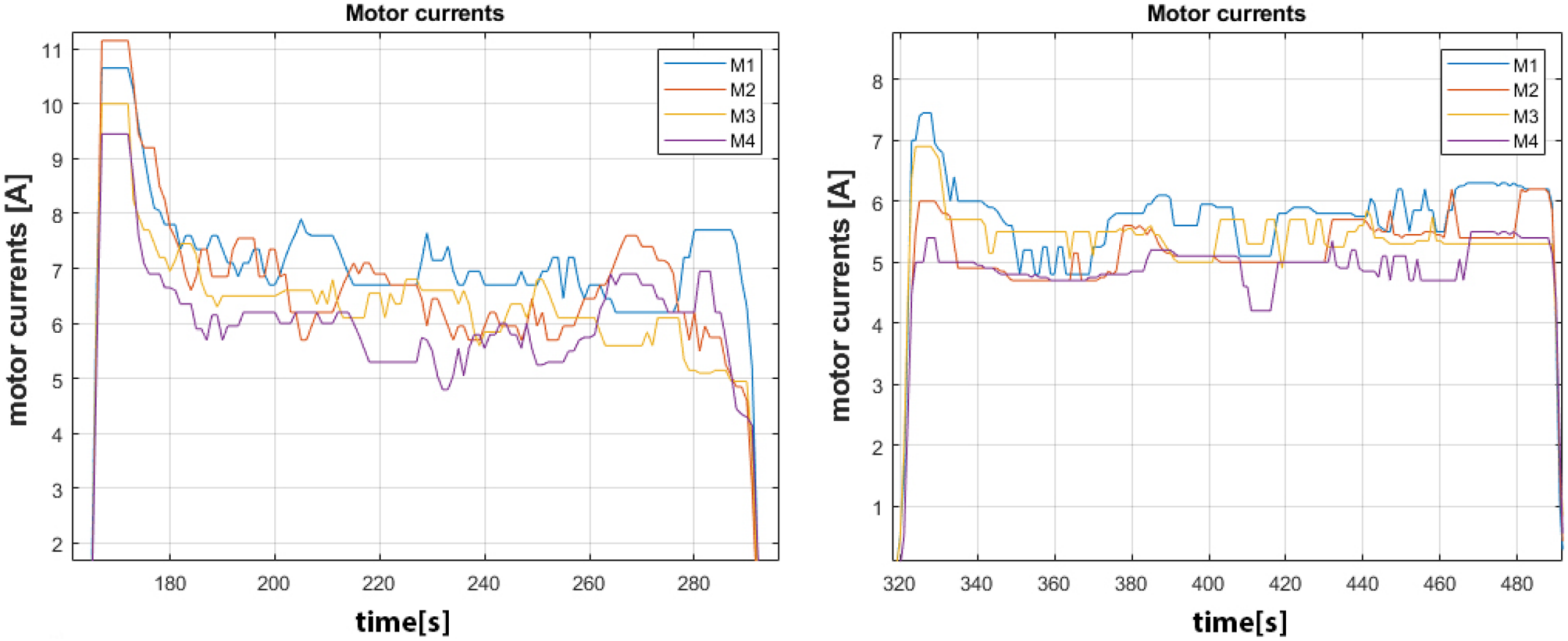
Currents drawn by motors over asphalt, on the left, and over concrete, on the right, during straight motion.

**Figure 12:**
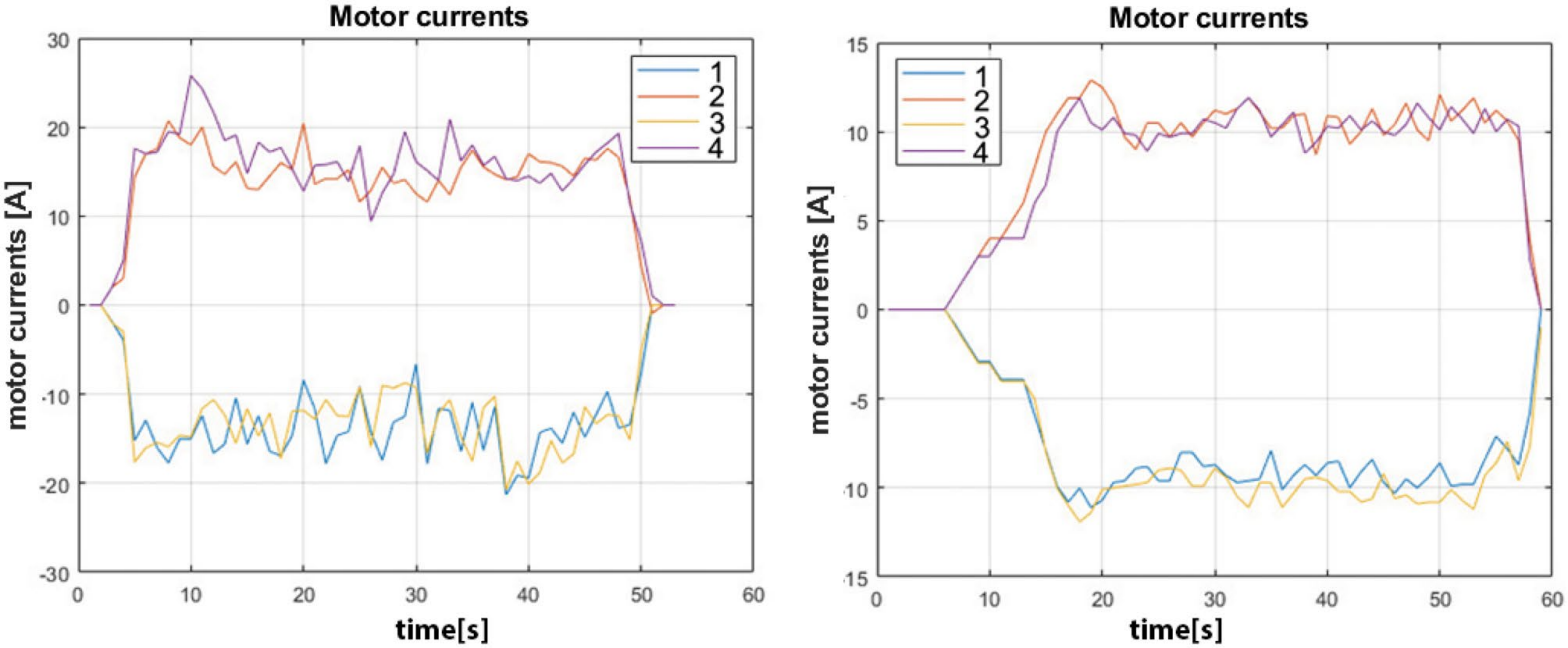
Currents drawn by motors during sideways motion on asphalt, on the left, and on concrete, on the right.

As reported in Fig. 10, the straight forward motion is obtained by making all the wheels spin in the same direction and by setting a fixed linear velocity, while, in order to make the vehicle move sideways, it is necessary to control motors M1 and M3 to make them spin clockwise and motors M2 and M4 to spin counter-clockwise at fixed velocity. Figure 11 highlights the motor currents drawn by the drive motors where time, expressed in seconds, is represented horizontally on the X-axis and the current amplitude, in ampere, is reported on the Y-axis, while the vehicle moves on asphalt (left) and industrial concrete floor (right) during straight motion. Since asphalt has a higher friction component and unevenness level than concrete, the average currents are about 6.5A with some maximum peaks above 10A when time t=170s during the transient starting stage. At steady-state conditions (between t = 180s and t = 277s), fluctuations in the electrical currents ranging from 6A to 7A can be noted due to asphalt irregularities and to sporadic contact loss of the omni wheels. On the other hand, average currents for concrete are about 5.4A at steady-state with maximum peaks at time t= 327s where they do not exceed 7A. The currents in the time interval between t=340s and t=460s fluctuate in quite a restrained way since concrete features a flatter and regular surface. It is very interesting to note that the sideways motion requires more power from the motors due to the sliding of the rollers placed at $45^\circ$ and the ground as shown in Fig. 12. For this reason, the average current drawn by the motors for sideways motion on concrete is 10.4A with a constant trend characterized by limited fluctuations from 9.5A to 11A in the time interval between t = 22s and t = 55s. On asphalt, the average current is about 16.6A with a maximum peak of 25A at t=10s and a more fluctuating behavior in the time interval between t = 10s and t = 45s because of the irregular surface as described for the forward motion. Moreover, during sideways motion, the currents of motors M2 and M4 are positive while the currents of motors M1 and M3 are negative since the vehicle is sliding on the left following the configuration reported in Fig. 10. All the plots report some spikes due to the intrinsic nature of the MDC2460 motor controller used for Omnibot and the 500W DC brushed motors.

**Figure 13:**
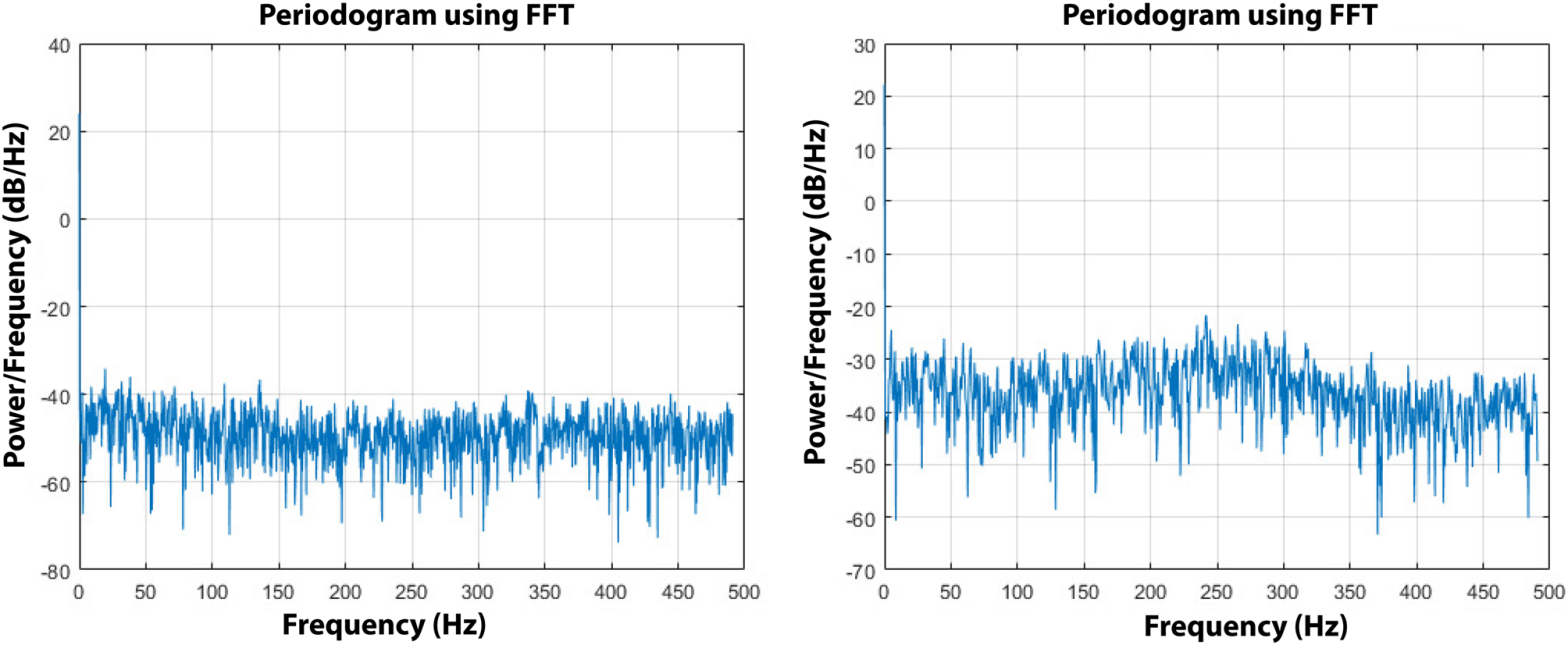
Periodogram from vertical accelerations over concrete, on the left, and over asphalt, on the right, during straight motion.

A second test case has been devoted to evaluate the robot vibrational response, as described in section III.D. Figure 13 shows two periodograms obtained as result of the vibrations over the vehicle’s frame on two different surfaces, concrete and asphalt, as an estimation of the spectral density of the vertical accelerations by examining the amplitude against the frequency characteristic measured by the inertial sensor. More details on the use of the power spectral density can be found in previous research studies^36–38^.

**Figure 14:**
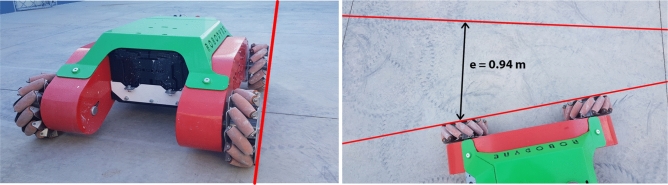
The vehicle positioned at the starting point on concrete, on the left, and the final error at goal, on the right.

**Figure 15:**
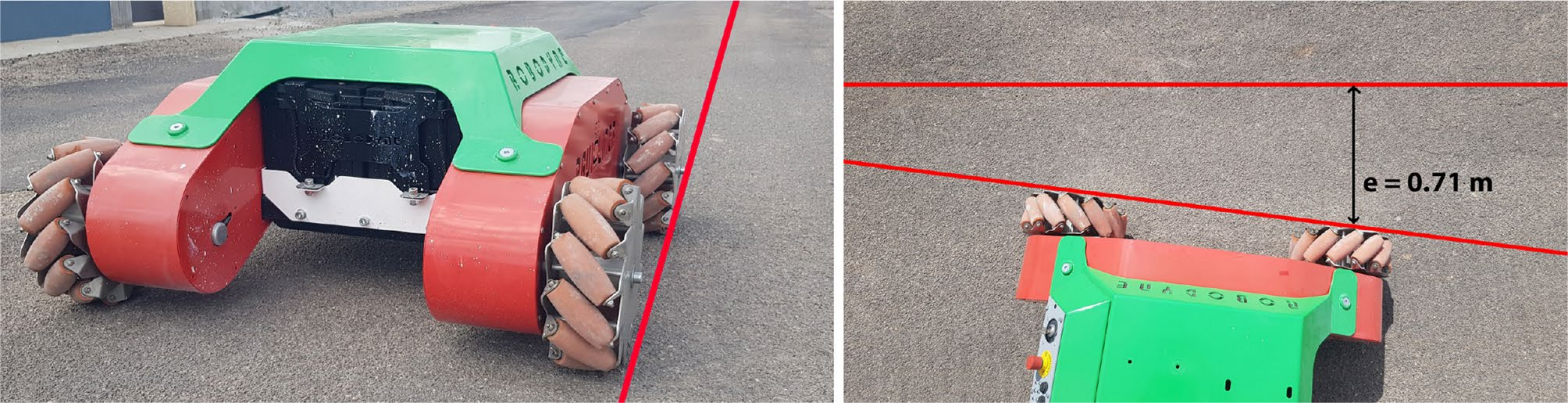
The vehicle positioned at the starting point on asphalt, on the left, and the final error at goal, on the right.

The average noise power per unit of bandwidth for the industrial concrete floor is about -49.29 dB/Hz while the average for the asphalt is -36.54 dB/Hz and this is because the asphalt provides a rough surface with small debris that contribute to increase the vibration response compared to the concrete floor that can be considered as totally flat. The features of the ground surface also have effects on the locomotion control of the vehicle since they introduce unpredictable slipping effects and slide motions that disturb the vehicle’s trajectory. A straight line reference made by a laser pointer has been set up both on the industrial concrete floor and asphalt in order to study the performance of the trajectory control. The right side wheels of Omnibot have been aligned with the red line reference, and the vehicle has been commanded to move forward and backward following a straight path for 10 meters with an open-loop control both on industrial concrete floor and on asphalt, for ten times for each direction, in order to measure the total accumulated error between the red reference straight line and the final position of the vehicle as showed in Figs. 14 and 15.

Before each test, the inertial sensor has been initialized, calibrated and set to $\theta _0=0 \deg$ in order to be able to accurately measure the final deviation angle $\theta _c$ on the concrete and $\theta _a$ on asphalt.

**Table 4:** Errors and yaw angle deviation for each test.

Test	Dir	Surface	$\Theta _c$ deg	Error m	Surface	$\Theta _a$ deg	Error m
1	F	Concrete	4.56	0.90	Asphalt	3.10	0.64
2	F	Concrete	4.81	0.94	Asphalt	3.15	0.65
3	F	Concrete	4.69	0.92	Asphalt	3.41	0.69
4	F	Concrete	4.75	0.93	Asphalt	2.95	0.61
5	F	Concrete	4.56	0.90	Asphalt	2.98	0.62
6	F	Concrete	4.20	0.83	Asphalt	3.54	0.72
7	F	Concrete	3.98	0.79	Asphalt	3.26	0.67
8	F	Concrete	4.21	0.83	Asphalt	2.91	0.61
9	F	Concrete	4.56	0.90	Asphalt	2.97	0.62
10	F	Concrete	4.34	0.86	Asphalt	2.85	0.60
11	B	Concrete	4.75	0.93	Asphalt	2.80	0.59
12	B	Concrete	4.56	0.90	Asphalt	3.20	0.66
13	B	Concrete	3.97	0.79	Asphalt	3.50	0.71
14	B	Concrete	3.89	0.78	Asphalt	3.40	0.69
15	B	Concrete	4.21	0.83	Asphalt	3.50	0.71
16	B	Concrete	4.34	0.86	Asphalt	2.70	0.57
17	B	Concrete	3.95	0.79	Asphalt	3.10	0.64
18	B	Concrete	4.5	0.88	Asphalt	2.80	0.59
19	B	Concrete	4.43	0.87	Asphalt	3.30	0.68
20	B	Concrete	4.60	0.90	Asphalt	2.90	0.61

**Figure 16:**
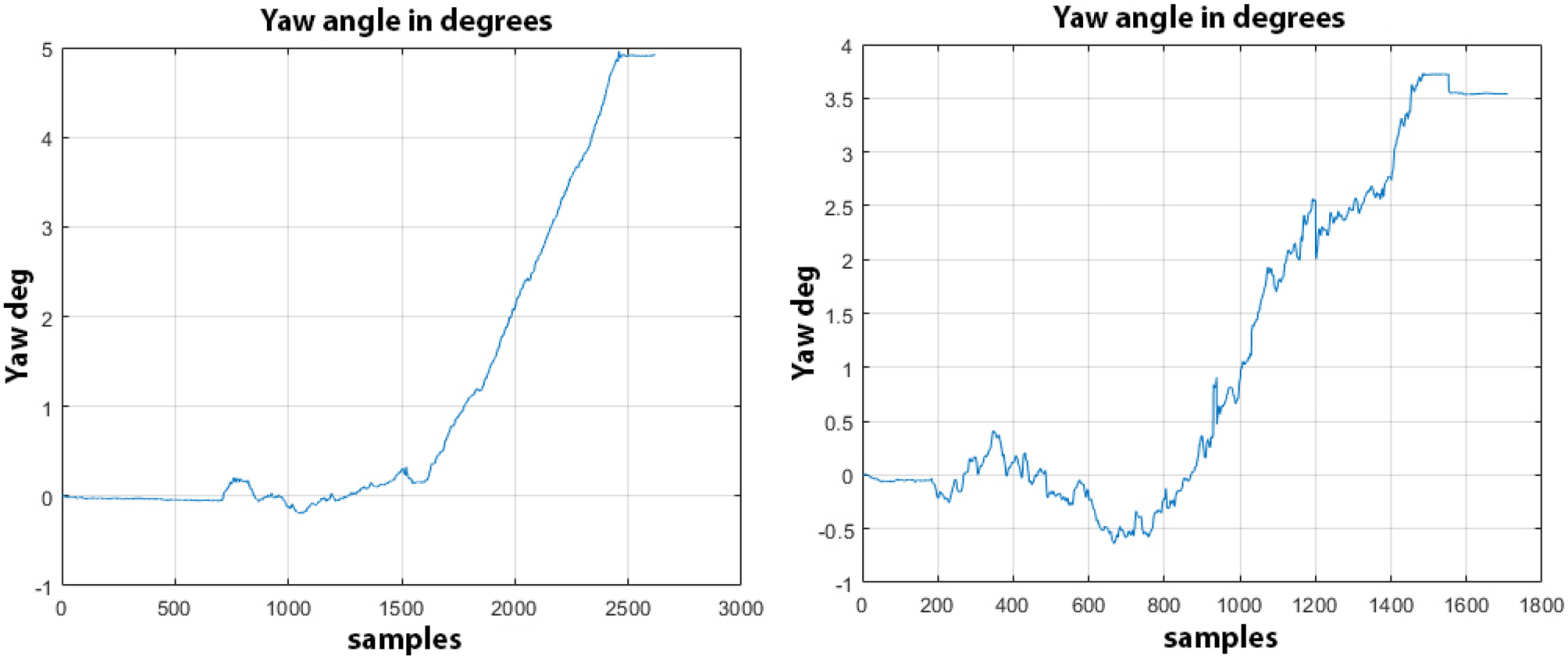
Angle deviation on straight trajectory on concrete, on the left, and the angle deviation for asphalt, on the right.

In all tests, the error has been measured thanks to a laser distance meter and the values are reported in Table 4 where it is possible to see how to maximum error has been registered during test 2 on concrete with $e_c=0.94 m$ and a maximum deviation angle $\theta _c=4.81\deg$ as highlighted in Fig. 16 where the Y-axis reports the values of the yaw angle expressed in degrees and the X-axis reports the samples acquired from the inertial sensor at 25Hz while the maximum error obtained on asphalt during test 6 is $e_a=0.72 m$ with a total deviation angle of $\theta _a=3.54\deg$.

It is worth noticing that the holonomic vehicle performs differently depending on the ground surface: when it is commanded to follow a straight line on industrial concrete floor, the slipping effects caused by the flat surface are higher than the ones introduced by the asphalt surface which, on the other hand, provides a more rougher surface with an higher friction coefficient that allows the rubber rollers from the mecanum wheels to have more grip by minimizing the skidding effects. The average difference between the angle deviation on concrete and on asphalt is more than $A_{\theta _c} - A_{\theta _a} = 1.3 \deg$ which is particularly relevant for longer trajectories since, for example, a difference on the angle deviation of just $1.3 \deg$ can lead to a total final error at the goal of about 2.37 m. By taking into account that both the average currents drawn by the motors and the PSD values can be considered as unique fingerprints for each ground surface^39^, it is possible to apply a closed-loop control as already described in Eq. (28) where the parameters $A_c$ and $B_c$ are generated by the ground classifier and are used as adjustment values to make the PID control outputs the motors corrections faster by avoiding waiving loitering around the track; the PID control has been tuned by running an experimental trial-and-error method based on a total of ten tests for each surface and by measuring each time the total final error. In the end, the PID control has been configured to work with $K_p=1.0$, $K_r=0.02$, $K_d=0.1$ and $A_c=1.1$ and $B_c=1.6$.

**Table 5:** Final errors and yaw angle deviation for each test.

Test	Surface	$\Theta _c$ (deg)	Error (m)	Surface	$\Theta _a$ (deg)	Error (m)
1	Concrete	0.38	0.17	Asphalt	0.52	0.19
2	Concrete	0.39	0.17	Asphalt	0.57	0.20
3	Concrete	0.32	0.16	Asphalt	0.56	0.20
4	Concrete	0.31	0.15	Asphalt	0.49	0.19
5	Concrete	0.40	0.17	Asphalt	0.41	0.17
6	Concrete	0.29	0.15	Asphalt	0.39	0.17
7	Concrete	0.34	0.16	Asphalt	0.54	0.19
8	Concrete	0.37	0.16	Asphalt	0.53	0.19
9	Concrete	0.34	0.16	Asphalt	0.57	0.20
10	Concrete	0.39	0.17	Asphalt	0.43	0.18

**Figure 17:**
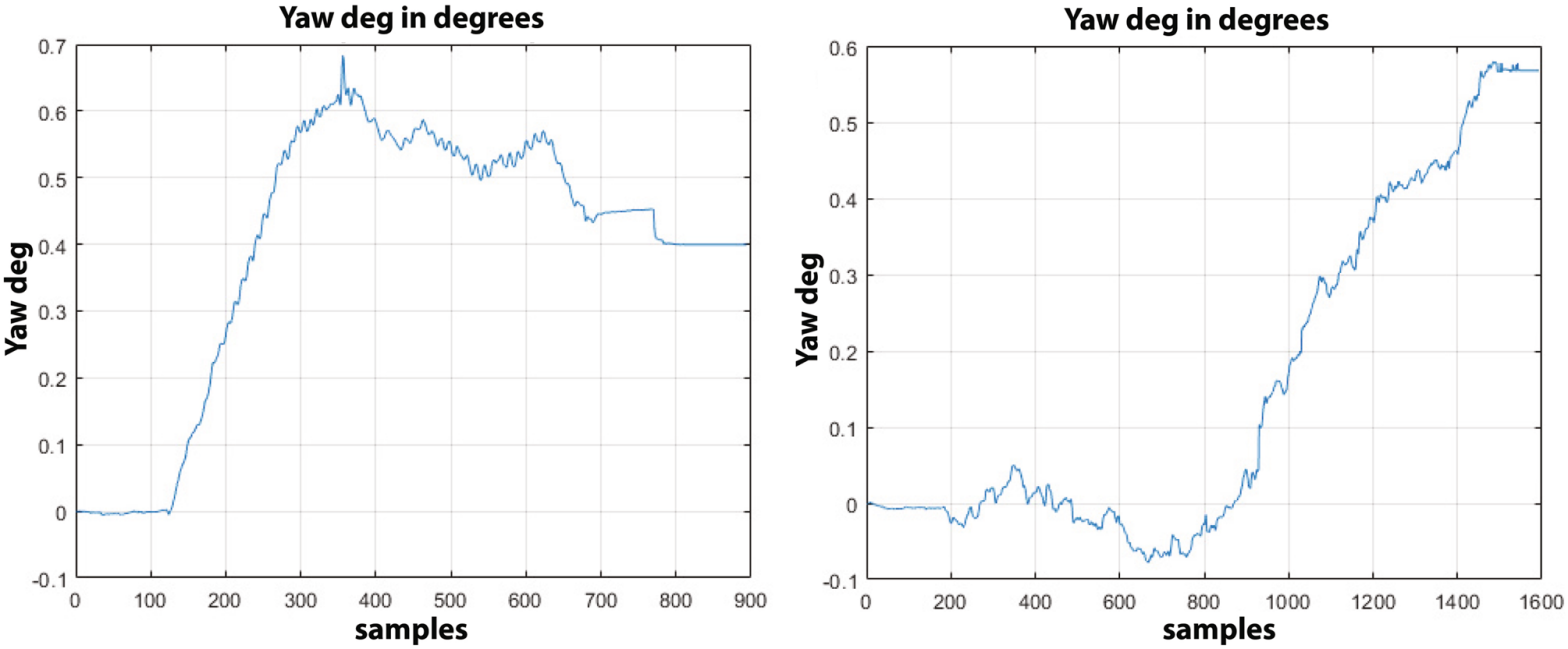
Final angle deviation with PID on straight trajectory on concrete, on the left, and the angle deviation with PID for asphalt, on the right.

**Figure 18:**
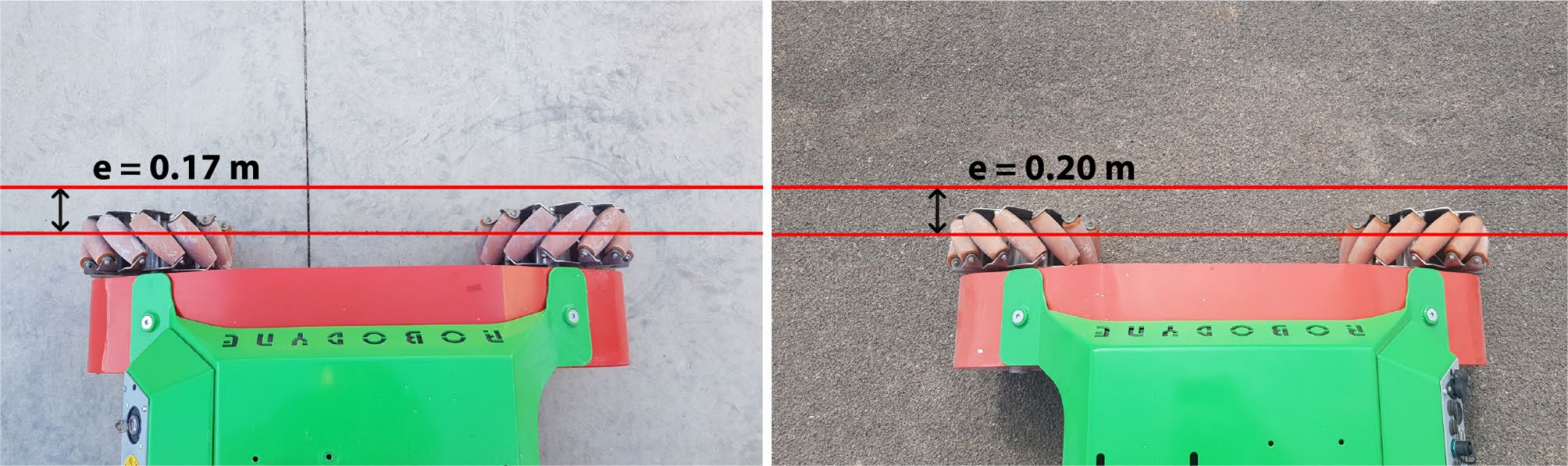
Final maximum error obtained on concrete, on the left, and on asphalt, on the right.

As reported in Table 5, by applying the closed-loop control, it is possible to make the vehicle keep on its track with a final maximum angle deviation of $0.40 \deg$ on concrete with a maximum final error of 0.17 m and 0.57$\deg$ on asphalt with a maximum final error of 0.20 m as reported in Figs. 17 and 18; moreover, it is interesting to note that on the concrete, there is typically an overshoot of about $+0.2 \deg$ caused by the derivative control of the PID while trying to minimize the slipping effect while the overshoot is almost null on asphalt because of its rougher surface.

## Load influence

**Figure 19:**
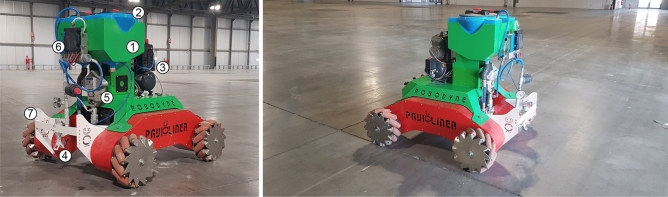
Omnibot equipped with a spraying unit, on the left, and the vehicle during the tests in the Fieramilano area in Rho, Italy, on the right.

One of the possible industrial applications of Omnibot is the automatic floor marking of large exhibition halls where the boundaries of the single exhibition booths need to be clearly set before the event. To this aim, Omnibot is equipped, as shown in Fig.  19, with an airless spraying unit powered by an hydraulic pump (5), commanded by a motor driver (6), that sucks out the paint stored in two tanks (2), placed in the upper rack (1), and feeds it to a bottom nozzle (4) usually placed at 25 cm away from the floor even if its height can be adjusted by using the positioning holes available on the nozzle support (7). Finally, an internal 12 VDC air compressor (3) is used to enable or disable the nozzle. The total weight of the sprayer unit is 94 kg resulting in an increase of about 50% of Omnibot original weight. An experimental campaign with a total of five tests was performed by commanding the vehicle to follow a 30 m straight line moving on a concrete surface inside the Fieramilano area in Rho, Italy. The proposed adaptive closed-loop control algorithm was adopted in all tests. The average registered errors of 0.15 m (worst case 0.18 m) and 0.34 deg (worst case of 0.42 deg) on the final position error and yaw deviation angle, respectively, fulfill the requirement for the automatic floor marking task. Performance can be seen in line with the previous results obtained from Omnibot without any payload, indicating that the adaptive control shows a high robustness to change in the payload.

## Conclusion

This research presented Omnibot, an omnidirectional vehicle intended for heavy duty industrial and service applications featuring large payload and increased energy autonomy by addressing specific issues including variability of the wheel radius and accurate pose estimation even in the presence of unavoidable wheel slip occurrences. A Kalman Filter-based pose estimation system working in conjunction with a classifier able to identify the ground surface has been used to retrieve the position of the vehicle, and a PID control has been tuned to generate the motors command in order to keep the vehicle on its track. First, the vehicle is operated in an open-loop configuration. In this case, the path is affected by external disturbances including noise, wheel slipping, surface unevenness and surface type. Then, a PID closed-loop controller is proposed with a feedback loop that continuously sends correction information by relying on the inertial sensor and on the PID parameters that are issued by the ground classifier and are used as adjustment values to make the controller generate the motors corrections faster by avoiding the waiving loitering around the track. The use of the closed-loop controller resulted in higher positioning accuracy and in the ability to react immediately to possible disturbances. The closed-loop approach resulted in a reduction of about 77% and 88% of the position and orientation error, respectively. The experimental results demonstrated that the proposed system is able to provide a final maximum angle deviation of 0.40$\deg$ with a maximum final error of 0.17 m along a 10-m straight path on concrete and of $0.57 \deg$ with a maximum final error of 0.20 m along a 10-m straight path on concrete. The performance can be further improved if laser detection sensors are used both to implement obstacles avoidance routine and to better retrieve the vehicle’s position by using static references such as walls, pillars, and fixed structures. The adoption of a suspension system for the mecanum wheels will be also investigated along with the implementation of other advances control strategies like the model predictive control (MPC) and the slide model control (SMC). By improving the wheel compliance, surface irregularities may be better accommodated resulting in a lower impact of the ill-effects due to wheel slippages and in a improved and more precise control of the robot.

## Data Availability

The datasets used and/or analysed during the current study available from the corresponding author on reasonable request.
